# Perceptions of Malawian Nurses about Nursing Interventions for Malnourished Children and Their Parents

**DOI:** 10.3329/jhpn.v29i6.9898

**Published:** 2011-12

**Authors:** Magdalena Johansson, John L.Z. Nyirenda, AnnaKarin Johansson, Birgitta Lorefält

**Affiliations:** ^1^Department of Medicine and Health Sciences, Division of Nursing Science, Faculty of Health Sciences, Linköping University, Linköping, Sweden; ^2^Embangweni Mission Hospital, Mzimba, Malawi

**Keywords:** Child, Child nutrition disorders, Health education, Interventions, Nutrition education, Perceptions, Phenomenography, Malawi

## Abstract

In developing countries, malnutrition among children is a major public-health issue. The aim of the study was to describe perceptions of Malawian nurses about nursing interventions for malnourished children and their parents. A qualitative method was used. Data were collected and analyzed according to the phenomenographic research approach. Twelve interviews were performed with 12 nurses at a rural hospital in northern Malawi, Southeast Africa. Through the analysis, two major concepts, comprising four categories of description, emerged: managing malnutrition today and promotion of a favourable nutritional status. The categories of description involved identification and treatment of malnutrition, education during treatment, education during prevention, and assurance of food security. The participating nurses perceived education to be the most important intervention, incorporated in all areas of prevention and treatment of malnutrition. Identification and treatment of malnutrition, education during treatment, education to prevent malnutrition, and assurance of food security were regarded as the most important areas of intervention.

## INTRODUCTION

In developing countries, malnutrition among children is a major public-health issue. It is one of the most serious global risk factors for illness and death (1,2). Malnutrition during childhood has an impact later in life as it is associated with significant functional impairment, reduced work capacity, and decreased economic productivity. Malnourished children are more likely to suffer from delayed psychological development, poor school performance, and lower intellectual achievements. In developing countries, 39% of children, aged less than five years (under-five children), are estimated to be chronically malnourished (2). Moreover, stunting, severe wasting, and intrauterine growth restriction together are responsible for 21% of deaths of under-five children worldwide (3). The rate of mortality among children with severe malnutrition is over 20% (3). In Malawi, the prevalence of malnutrition—defined as weight or height that falls more than two standard deviations below the normal—among children is estimated to be 49% (4,5). Despite this, enough focus has not still been given on interventions and treatment to promote a better nutritional status among children in developing countries (6).

Malnutrition does not only seem to be a poverty issue. Lack of knowledge and low dietary quality of food given to children are also two likely contributing factors (7,8). There is some evidence that nutrition-education interventions can result in the favourable growth and development (8). Nutrition is an important part of nursing care. Good healthcare cannot be provided without having satisfactory knowledge about nutrition (9,10).

This study endeavours to describe how the nurses at a rural hospital in Malawi thought of the issue, how they felt that they could help malnourished children and their parents, and what they thought could be done to improve the situation further. The aim of the study was to describe perceptions of Malawian nurses about nursing interventions for malnourished children and their parents.

## MATERIALS AND METHODS

This is a phenomenographic study, which means that the researcher is interested in how phenomena are conceived by others, i.e. how other people describe their experiences of various aspects of the world (11-14). The interviewed nurses were chosen to represent a range in age and experience to cover as many aspects as possible of the phenomenon analyzed. Thus, a strategic selection process was used for finding participants that corresponded to the predefined plan of the study. According to the plan, the participants were required to perform nursing interventions and work with malnourished children either at the hospital or in primary healthcare. A range in age and experience was also desirable. The age of 12 nurses enrolled ranged from 25 to 60 years, and the years of experience ranged from two to 30 years. Five of the nurses were male, and seven were female; 50% worked in primary healthcare; and 50% worked in hospital-based care. Nevertheless, all the participants had some experience in primary healthcare and community-based care.

Data were collected in a rural area in northern Malawi during July-August 2009. During this period, 12 interviews with key-informants were performed. During data-collection, the interviewer considered aspects that could be stressful for the participants and tried to create an atmosphere that allowed an open dialogue. All the interviews took place in a separate room at the hospital with the door closed. Throughout all the interviews, the interviewer, who was independent from the nurses, was alone with the participants. The interviews were recorded. A pre-prepared semi-structured interview guide, consisting of open-ended questions, was used. The first questions in the interview guide were simple, and the general ones aiming at introducing the respondents to the subject and help them relax in the situation. During a qualitative interview, the interviewer has to make it clear that the participants can feel comfortable and be allowed to think out loud. The questions in the interview guide aim to encourage the participants to share descriptions of their experiences and need not be followed strictly; rather the dialogue should proceed according to the answers given by the participants (11). At the end of the interviews, the participants were given the opportunity to add more information about the subject if they thought that there was something they had not been able to mention earlier. The interviews were performed in English. When the interviews were finished, they were literally transcribed. One of the co-authors took part in transcribing the materials and contributed with re-evaluation of the analysis.

In the phenomenographic analysis, seven steps can be distinguished. These steps were followed in the path of analysis in the study. During the first step—*familiarization,* the transcribed interviews were carefully read and reread several times, and some errors in the text were corrected. Next step was the *compilation* where the parts of the text that contributed with significant perceptions of the phenomenon were identified and marked. Next, the perceptions were reduced into their essential parts in the step *condensation*. In the fourth step, the preliminary *grouping* or *categorizing* of similar answers took place. In the fifth step—*comparison*— the categories were compared with each other to establish evident borders between them. During the sixth step—*naming,* the categories were named to elucidate the essence of each category. In the seventh and last step, a *contrastive comparison* of the categories was made to find a description of the categories that clarified their essence and the resemblance between them (11,13). To describe the results of the analysis, the categories of description were organized in an outcome space to demonstrate the central meaning of distinct perceptions. They reflected the number of qualitatively different ways the phenomena had been described, understood, and analyzed (12).

### Ethical aspects

When designing and performing the study, the intentions of the Helsinki Declaration (15), ethical standard principles (16), and Nordic ethical rules (17) were followed. The local ethical committee in Malawi was consulted and approved the study. Participants were not identifiable in the database or in published material. Before the interviews were carried out, the participants were informed about the purpose of the study and that everything would be recorded but also that all would be confidential, and only the interviewer would listen to the recorded material afterwards. They were also informed that they had the right to interrupt the interview or withdraw from the study at any point of time without giving any reason. Furthermore, written consent was obtained from all the participants.

## RESULTS

The analysis resulted in an outcome space where 14 different types of perceptions about the phenomena were found. These perceptions were established as sub-categories of the four different categories of description. Subsequently, the categories of description were divided into two main categories (Fig.). All the categories were illustrated with quotations from the participants.

**Fig.**
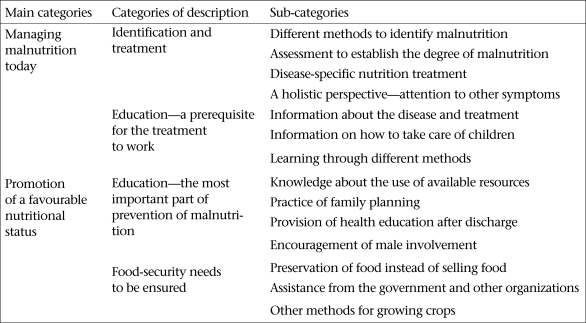
Perceptions of Malawian nurses about interventions for malnourished children and their parents

The nurses described the identification of malnourished children, defined as weight or height that falls more than two standard deviations below the normal, as an important part of their work but also as an uncomplicated task that was easy to do and that different methods and guidelines could be used for assisting them. These methods included, for instance, measurement of weight and usage of a chart with limits for underweight clearly marked. The weight was plotted in the chart to discrete those who were underweight. They also measured the height and calculated the relationship between weight and height according to the guidelines of the World Health Organization. Furthermore, they emphasized the measurement of mid-upper arm circumference (MUAC) which they thought was an important method to detect malnutrition, unless the children had developed oedema. They described oedema as an independent criterion for the detection of malnutrition. Following the identification of malnourished children, the participants described that it was necessary to assess the patient to determine the degree of malnutrition and to be able to decide what treatment was suitable for each patient. To provide the right treatment, the participants expressed the importance of noticing other symptoms or diseases besides the malnutrition. These could be fever, cough, dehydration, hypothermia, hypoglycaemia, anaemia, diarrhoea, or other infections.

We assess the child, head to toe, we assess the hair texture, and we assess the eyes … and then we also assess the abdomen and maybe he has got a descendent …. Besides, we also assess the extremities …. Then after assessing we find the diagnosis.

Telling the mother or the caregiver about the disease and educating them about the treatment was expressed as being the most crucial part of the treatment. The participants felt that, if the mother was able to take part in the treatment, her compliance would be increased. The nurses explained how they tried to educate the parents during their stay at the hospital.

But if she does not know about the treatment, she can just go away when it is the time for that milk, she can just move out.

The participants experienced that using different teaching methods was advantageous. They used pictures, songs, drama, and cooking-lessons. Some of them emphasized how they could encourage the parents to take part in education by showing them pictures. One nurse explained how she used a picture-cord in front of a group of mothers as an example of poor nutrition behaviour to help them understand the issue. The picture-cord demonstrated an example well-known to them; thus, they could relate to the situation. Furthermore, they were able to relate the situation on the picture-cord to their own knowledge, thereby understanding the essence in the message.

I can remember another time using a picture-cord where the father looked very heavy, and every good food was on his table. And the wife was pregnant, and she was sitting with the siblings on the floor and they were eating very little food …. And the mothers said, “what can we do to reverse the situation?” … in the end of the day, they would go to their households with the key message which they have created themselves.

A central issue in the education for the prevention of malnutrition was the use of available resources. The nurses felt that the rural people did not have enough money to buy food but that they were able to grow different types of foods to achieve an adequate food intake. Hence, they needed knowledge about what to grow and how to best use their crops.

Food resources … where they can find them … in our homes we have got different types of foods. We have got legumes, vegetables, fruits, groundnuts and those things, and all these are things that we can manage malnutrition with. So, we say: the little food we are having but try to source it up and try to give these to children.

The nurses found promotion of family planning to be a specially-important intervention to prevent malnutrition and that information about family planning was a substantial part of health education which they were supposed to give to the parents. They explained that the majority of mothers gave birth to children without adequate time between each child, and, therefore, the mothers could not give enough care to their infants. They also thought that breastfeeding was affected if the families had too many children. They mentioned cultural aspects and how ignorance could contribute to unfavourable breastfeeding practices during pregnancy. Thus, the nurses thought that education about breastfeeding was also an important issue and that this problem was another reason to provide education on family planning.

It is good to advise them about family planning because they are just having their children each year, they cannot be able to take care of their children.

The participants would like to use follow-ups as a form of intervention. Due to long distances and lack of both staff and transport, this is currently not an option. The hospital staff felt that they lost control of the ways parents feed their infants when they had left the hospital. Therefore, they felt that not being able to continue with the education after the parents had left the hospital was a problem. They also experienced a lack of compliance and more re-admissions as a result of no follow-ups.

Most children were just being discharged without making follow-ups; so, when they go home, we do not have an idea whether maybe they are taking the right amount of food or not.

The nurses explained that a lot of people lived very far from the healthcare facilities, and many children died as a result. The nurses felt that, if they went out to villages, they could better understand the type of problems people had to deal with and, thus, they could help them better.

Lately, we have established more mobile clinics with the aim of reaching out to these mothers … like here at the hospital they do not understand us but when we go closer to them they do.

The participants regarded interventions to promote male involvement as critical for the prevention of malnutrition. They implied that men rule the homes in Malawi, they have the money, and they do the decision-making. The nurses thought that it was complicated to cooperate with men regarding the health issues and that it was hard to encourage them to come to the hospital. The nurses felt that it was difficult for the woman herself to pass the information to her husband; he would not listen to her. One male nurse explained how he as a nurse could act as a good example for the husbands. By showing men that they too can care for children he thought that he could encourage the fathers to do the same.

If the women come with their husbands, we are open to their husbands, sitting down, “look husband we are here, we are men, and we are men taking care.”

The nurses experienced that it was a problem that people were not able to make the food last for a whole year. They described that people often sell their food instead of saving and preserving it and that the result of such practices was that the food would be finished long before the people could harvest again. To ensure food security, the participants thought that cooperation with other organizations would be beneficial. They also described different interventions that they thought the Government could carry out to assist the people. Through such cooperative interventions, they thought that better practices could come about and that development of their country could take place. The nurses regarded themselves as an important part of these developments but they also stated that they could not manage it by themselves. They felt that each instance in the society could contribute to a favourable development.

## DISCUSSION

In the results, education emerged as the most important intervention for malnourished children and their parents. Different types of education targeting different areas were mentioned. Education could be seen in all parts of interventions for malnourished children and their parents. Hotz and Gibson described how nutrition-education programmes could increase the ability of mothers to provide their children with nutritious food, which corresponds to the results in this study (8). As in other studies, intensive nutrition education has been shown to reduce malnutrition and mortality among malnourished children. Therefore, education can be used as a prevention and management strategy in long-term nutrition practice in communities in developing countries (18).

Using different methods when educating parents can be beneficial to make them understand the subject. Hubley discussed health education in developing countries in his article (19). His conclusion was that development of planned programmes with different types of educational methods, including clear and simple advice, is some key ingredients for success. Hubley mentioned, for instance, pictorial advice, participatory learning methods, and practical demonstrations as adequate methods for education of patients in developing countries. His view of education corresponds well with perceptions of our study participants.

The results of this study showed that emphasizing the importance of education on family planning may have an advantage to the health of children. Studies have shown that fertility ideas and sexual behaviours of people are influenced by the behaviours and attitudes of others in their social groups. Therefore, good examples and widespread knowledge are crucial for changing attitudes and promote practices of family planning (20). A link between breastfeeding and family planning was found in our study. The study participants felt that breastfeeding practices were not optimal in families that did not follow family planning. Some participants felt that ignorance could lead to inappropriate practices of family planning and as a consequence inappropriate breastfeeding, which is consistent with results of the study by Vaathera *et al*. (21). Results of several studies suggest that breastfeeding is an important issue in the struggle against malnutrition and death among children (21-23). In Malawi, when a child is weaned from breastfeeding, he or she is often sent to stay with the grandmother.“This change”of environment contributes to loss of appetite of the child and hence malnutrition.

Problems with male involvement in the health issues were another interesting aspect of indirect causes of malnutrition. Since men are often the ones who make decisions in families, they play an important role in the nutritional conditions in a household. This can also be found in other studies from different parts of the world, which implies that the problem is widespread and that promotion of male involvement in the health issues should be a critical issue to work with (24).

Another result in our study was that the healthcare needs to make an effort to mobilize its resources to assure that knowledge and healthcare are accessible for everyone. In many cases, relapses occurred when the children went back home after receiving treatment for malnutrition. This indicates that there is a need to continue providing the parents or other caregivers with knowledge about how they can feed and take care of their children properly, which is also stipulated by Sangala (5). In this study, the participants expressed a wish to improve their routines for follow-ups. Follow-ups have been viewed as a good way to improve the health status in developing countries (19). Different methods to reach out to people in communities have been discussed. Charlton *et al*. found that a programme aimed at training healthcare workers in villages did not have beneficial effects on the nutritional status of children (25). This result does not correspond to the results of the present study, which showed that all education appeared to be advantageous. However, it is interesting to note the importance of evaluation of different educational methods that Charlton *et al*. indicated in their study (25), which the participants in the present study seemed not to consider.

The phenomenography method is mainly aimed at describing variations of a phenomenon and the different ways people can experience it (11-14). Hence, the approach could be regarded as useful in the present study as there was an ambition to describe perceptions of nurses about a specific phenomenon. The seven steps that were followed in the analysis helped the authors follow a consistent path during the analysis without missing important parts. A weakness of the phenomenography approach is, for instance, the never-ending reversion of the categories. It is often possible to find another system of categories to describe perceptions of participants better (11). In the present study, the categories were revised several times to find the optimal way of describing the phenomenon. The trustworthiness of the study was also enhanced by the re-evaluation of the analysis made by one of the co-authors. Additional limitation of the study was the language barrier as English was neither the authors’ nor the participants’ native language.

In an interview situation, it is important that the participant can feel comfortable and be able to speak form his or her mind (11). The ambition of the interviewer was to create an open and permissive atmosphere. All the interviews were performed in a separate, closed room, and the only people present were the author and the participant. Starting with some simple general questions was also a way of helping the participants to relax. The fact that the place was well-known to the participants could also have advantages. It is always a risk that the participants consider it important to answer the questions in a correct way to show their competence and perhaps have some benefits from the author in the future. Nevertheless, the answers of the participants were perceived sincerely, and it was, thus, not regarded as a major limitation of this study.

The phenomenographic approach has several advantages when applied to healthcare and nursing research. It can describe aspects that cannot be investigated by quantitative methods, and it emphasizes the different ways patients, or health workers, experience healthcare. Such knowledge can help healthcare professionals to develop evidence-and individual-based care for each patient (11,12). Considering the extent of the present study, the findings cannot be generally applied in the care for malnourished children and their parents but it can inspire to build a foundation for quantitative research in the field.

### Conclusions

The findings of the study describe how the participants strove to target different areas for treating and preventing malnutrition. Education was perceived as the most important area by the participants. They pointed out that this is a critical intervention in every step in the treatment of a malnourished child, often performed with the help of different pedagogical methods. The care for malnourished children is multifaceted and requires special nursing skills and a holistic perspective of children and their parents. The nurses described how different methods and guidelines and also cooperation with other professionals could help them in the process of treatment and prevention of malnutrition.

## ACKNOWLEDGEMENTS

The study was funded by the Swedish International Development Agency, which has had no role in the design or performance of the study. The authors wish to express gratitude to the participating nurses who shared their time and experiences.
